# IL-4, TSLP and IL-31 Cytokine Profiles as Related to Psychometric Measures in Patients with Mastocytosis

**DOI:** 10.3390/ijms26020529

**Published:** 2025-01-09

**Authors:** Jan Romantowski, Kinga Fabiańczyk, Maria Skrzypkowska, Wiesław J. Cubała, Piotr Trzonkowski, Marek Niedoszytko

**Affiliations:** 1 Department of Allergology, Medical University of Gdansk, 80-210 Gdansk, Poland; kingabojahr@gumed.edu.pl (K.F.); mnied@gumed.edu.pl (M.N.); 2 Department of Medical Immunology, Medical University of Gdansk, 80-210 Gdansk, Poland; maria.skrzypkowska@gumed.edu.pl (M.S.); ptrzon@gumed.edu.pl (P.T.); 3 Department of Psychiatry, Medical University of Gdansk, 80-210 Gdansk, Poland; cubala@gumed.edu.pl

**Keywords:** mast cells, psychometric measures, urticaria pigmentosa, bone marrow

## Abstract

Mastocytosis is a rare neoplastic disease of the bone marrow. Common symptoms like urticaria, diarrhea, bronchspasm and flushing are caused by mast cell degranulation and are mostly based on mast cell mediator release and Th2 type inflammation that occurs frequently in these patients. Psychological disorders are more prevalent in patients with systemic mastocytosis, though little is known about the mechanism behind this. The aim of the study was to investigate the Th2 cytokine (IL-4, TSLP, IL-31 and IL-33) profile in patients with mastocytosis in relation to classic degranulation symptoms and the psychometric measures of cognition and distress symptoms.In total, 115 patients diagnosed with mastocytosis were enrolled. Mini-Mental State Examination (MMSE) was performed for all subjects. Other variables: Quality of life in mastocytosis, a mood assessment commonly used in systemic mastocytosis by a certified rater—the Hamilton-17 Depression Scale, Pruritus Visual Analog Score, serum tryptase concentration and bone marrow biopsy results (archival) were also analyzed/included. Serum concentrations of IL-4, TSLP, IL-33 and IL-31 were analyzed as primary outcomes. For comparison with continuous variables linear regression was used. The mean MMSE result was 27.9. Regression analysis did not reveal significant correlation between the IL-4 (*p* = 0.82), IL-31 (*p* = 0.24) and TSLP (*p* = 0.37) serum concentrations and MMSE. The IL-33 concentration analysis resulted in 0 for all patients (was not detected). No significant effect was observed with other endpoints as well. One in four patients with mastocytosis presents cognitive decline. This impairment does not correlate with Il-4, TSLP, nor IL-31 serum protein concentrations.

## 1. Introduction

Mastocytosis is a rare neoplastic disease of the bone marrow [1]. Its key feature is the uncontrolled proliferation of mast cells which may affect different organs. The excess usually affects the skin, bone marrow, liver, spleen and gastrointestinal tract. If the disease is located solely in the skin, skin mastocytosis is diagnosed. If other organs are affected, the systemic version of the disease is diagnosed. Skin mastocytosis is commonly observed in children and usually presents a less severe course and might even disappear as the child grows up [2,3,4]. On the other hand, systemic mastocytosis is typical in adults and is associated with a greater variety of manifestations from different organs and might progress to an aggressive form of the disease. The symptoms of mastocytosis include: (1) those caused by mast cell degranulation; for example, urticaria, diarrhea, bronchospasm, rhinitis, anaphylaxis and headaches; (2) those caused by pathological mast cell infiltration in aggressive mastocytosis (ASM) with the destruction of the affected organs—typically the bone marrow and liver [5,6,7,8,9]. Fortunately, the vast majority of adult patients with mastocytosis suffer from Indolent Systemic Mastocytosis (ISM), which is usually associated with multiple degranulation symptoms, but is less life threatening and life expectancy remains standard.

The cognitive performance of an individual involves basic human skills such as attention, short- and long-term memory, communication, orientation, reading and calculating [10]. These typically deteriorate with aging; however, a sudden drop could suggest cognitive impairment. The Mini-Mental State Examination (MMSE) is a commonly used screening questionnaire for assessment of cognitive functions and presents the result in points (from 0 to 30) [11]. It is mainly focused on writing and reading; thus, in highly literate populations (such as most of Europe) the recommended uncorrected cut-off point for cognitive impairment is below 24. However, it is suggested that scores of 24–26 should be considered as Mild Cognitive Impairment (MCI) [11].

It is already known that cognitive disfunction and mood disorders are more prevalent in patients with systemic mastocytosis [12]. The prevalence varies greatly from 7% up to 70%, mostly due to different tools used for cognitive evaluation. In a study using MMSE, 7% of cognitive impairment was detected while 24% of patients presented MCI [13]. Another study using the Continuous Performance Test, Digit Span and Wechsler Memory Scale detected a 70% impairment rate in memory function and sustained attention [14]. Some of those reports highlight the fact that these cognitive impairments of patients could be reversed by disodium cromoglycate. This would suggest that these symptoms result from mast cell degranulation rather than aggressive infiltration or neurological destruction [1,9]. However, it is still unclear which mast cell mediators contribute to this factor. Symptoms such as diarrhea, urticaria and bronchospasm result mostly from histamine release. Other symptoms result from different mediators; for example osteoporosis and fever—from IL-6, heparin and tryptase, or fatigue and weight loss—from TNFalfa [15,16,17]. In addition, it has been found that mast cells take part in multiple neurodegenerative diseases [18]. In a murine model, mast cell-deficient mice show less severe multiple sclerosis. In Alzheimer’s disease, mast cells influence amyloid concentration and show an increased presence in brain tissue. In stroke, mast cells are found in neuroinflammation and tissue edema.

ISM is rarely considered to be a life-threatening disease, though it strongly impairs quality of life. Psychological disorders are one of the most important contributors to this problem [12,19]. In depression, a few studies have addressed the problem with treatment that targeted primarily mast cell degranulation symptoms. Lortholary et al. measured the Hamilton-17 Depression Scale (HDRS) in a randomized, placebo-controlled trial with the tyrosine kinase inhibitor (TKI), masitinib. This revealed a 75% improvement in depression and fatigue, though surprisingly this change was statistically non-significant [20]. Another TKI, avapritinib, used on patients with advanced mastocytosis, caused improvement in 75% of patients and total remission in 36%. However, in 30% of patients, new cognitive disfunctions appeared. This side effect might be due to a certain functioning alteration in mast cell degranulation [21].

In the recent literature, Th2 cytokine inflammation is suspected to play an important role in mastocytosis symptoms, including psychological [22,23,24]. IL-4, IL-31 and IL-33 are suspected to act together in mast cell homeostasis and secretion [24,25,26]. In particular, Il-4 and IL-31 are suspected to play a part in depression [27,28]. Additionally, Thymic Stromal Lymphopoietin (TSLP) acts together with IL-33 and also activates an abnormal mast cell for tryptase production in mastocytosis [29,30]. These findings regarding the role of the Th2 cytokine in mastocytosis symptoms might also show vital in search of anti-symptomatic treatment in the future as new biological drugs targeting those cytokines were recently developed [31]. Current anti-mediator therapies in mastocytosis are scarce and new biological additions would be of great help for patients.

The aim of the study is to investigate the Th2 cytokine profile (IL-4, TSLP, IL-31 and IL-33) in patients with systemic mastocytosis in relation to classic degranulation symptoms and psychological symptoms.

## 2. Results

The total number of recruited patients was 115, aged 19–79 (Mean 51). A total of 85 patients were women and 27 were men. The most prevalent type of mastocytosis was Indolent Systemic Mastocytosis (n = 76). Other patients presented Maculopapular Cuteneous Mastocytosis (MPCM; n = 15), Bone Marrow Mastocytosis (BMM; n = 8), Mast Cell Activation Syndrome (MCAS, n = 5), Mastocytosis in the Skin (MIS, n = 4), Mastocytosis Associated with Hemalotogic Neoplasm (M-AHN, n = 3), Smoldering Systemic Mastocytosis (SSM, n = 2), Mastocytoma (n = 1) and Diffuse Cutaneous Mastocytosis (DCM, n = 1).

Out of the 115 recruited patients, 30 (26%) patients presented cognitive impairment: 9 with a score below 24 (7.8%) and 21 (18%) with MCI. The mean value of MMSE was 27.9 (STD dev 2.62). The mean tryptase level was 48 ng/mL (Std dev 47.49), ranging from 2 ng/mL up to 214 ng/mL.

In depressive mood analysis with HDRS, the mean result was 9.98 points (STD dev 7.08) [29]. When assessing anxiety with STAI-S, the mean was 40.75 (STD dev 11.15). According to the Kayikcioglu et al. classification, 44 (38%) patients presented no state anxiety, 28 (24%) moderate anxiety and 43 (37%) presented high anxiety [32]. When taking into account trait anxiety with STAI-T, the mean was 43.56 (STD dev 10.52). A total of 40 (35%) patients presented no trait anxiety, 22 (19%) patients presented moderate anxiety and 53 (46%) high anxiety. The results of the baseline psychological evaluation of the studied group are presented in Table 1.

For the main symptom, pruritus, the mean Visual Analog Scale (VAS) result was 35 mm/100 mm. The mean QLMS was 46.77 points out of 120 points (Std dev 21.47).

**Figure 1:**
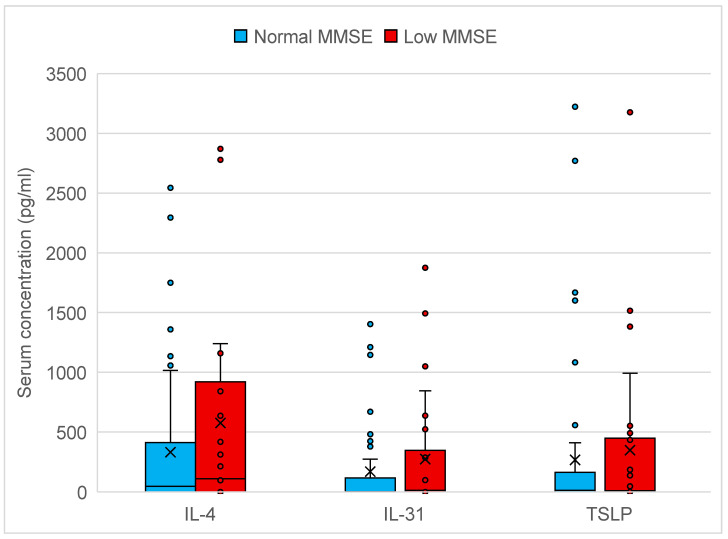
Comparison of serum concentrations of selected cytokines IL-4, IL-31 and TSLP and results of MMSE according to cut-off point <27 points (inclusive). MMSE—Mini-Mental State Examination. The differences are not significant in logistic regression for IL-4 (CI: −0.001–0.003), IL-31 (CI: −0.003–0.003) and TSLP (CI: −0.032–0.001).

In 80 patients, cytokine profile analysis was conducted. Regression analysis did not reveal a significant correlation between IL-4 (*p* = 0.82 CI: −0.003–0.001), IL−31 (*p* = 0.24 CI: −0.003–0.001) or TSLP (*p* = 0.37 CI: −0.001–0.003) serum concentrations and MMSE. The cytokine profile did not significantly impact secondary questionnaires nor serum tryptase levels. The IL-33 concentration resulted in 0 for all patients (was not detected), thus the analysis was not performed. The results of cytokine concentrations and statistical analysis are presented in Table 2. Adjusting for sex and age did not reveal any changes to the coefficient nor confidence intervals. No relationship between the time of assessment (the study started in winter and ended in summer) and the endpoints was found. The patients were also divided according to commonly validated cut-off points for MMSE into two groups (positive and negative for cognitive disfunctions). The cytokine concentrations in both groups are presented in Figure 1.

## 3. Discussion

The main finding of our study was the lack of a significant connection between cognitive disfunctions and other psychological issues with selected Th2 cytokines. In general, the results of the cytokine concentrations were relatively low and, in many cases, under the detection threshold. According to that fact, the assessment of a particular cytokine activation might be difficult to judge by serum concentration. Still, visual analysis of Figure 1 shows some difference in the concentration of the tested cytokines in groups with cognitive disfunctions, however statistically insignificant. It is possible that the cytokine inflammation profile is better analyzed using mRNA or a combination of ELISA and mRNA. The cytokine protein levels in serum fluctuate greatly and might be stable only locally on a tissue level [33,34]. Thus, using an alternative or additional method of gene expression might provide confirmation of Th2 cytokine involvement in the cognitive disfunctions in mastocytosis.

Our study shows that according to MMSE approximately 26% of patients with mastocytosis present cognitive disfunctions and 8% present a score below 24. This is in line with the Spolak-Bobryk et al. results that suggested a 24% prevalence and used the same method for evaluation [13]. Moura et al. used a different approach, the third edition of the Clinical Memory Scale by Wechsler that concentrated on memory assessment [19]. Immediate auditory ability was impaired in 41% of patients and working memory in 73%. This discrepancy is probably due to different methods, though it might also suggest memory problems as the main cognitive disfunction in mastocytosis. Similarly to our study, cognitive impairment was not related to depression, suggesting a quite different mechanism in those two symptom sets. Anxiety was present in 59% of the patients. Despite that fact, the quality of life in the studied group was high and patients’ mean score was only 23. That might be the result of the fact that patients adapted well to their psychological problems during the long-lasting disease.

Twelve studies have assessed the prevalence of depression in mastocytosis. Similarly to cognitive skills, a large discrepancy of prevalence was revealed—from 12% to 78% [12]. These differences were probably caused by using different tools. The most commonly used tool for the assessment of depression in mastocytosis is the 17-item Hamilton Depression Rating Scale (HDRS). Although the HDRS is not a diagnostic tool for depression, it is still commonly used in mastocytosis for the control of patients’ mood abnormalities that are present in the disease’s clinical manifestations [12]. Moura et al. evaluated the prevalence of depression with the HDRS at 68% while revealing the prevalence of cognitive impairment at 74%. It is worth noting that these two disturbances did not correlate with one another [19].

The problem with anxiety in mast cell disorders has been reported before. In a study by Nicoloro-SantaBarbara et al., the patients on average reported being “somewhat” anxious while 29% reported at least moderate anxiety, which is in line with our study [35]. It is known that in many chronic diseases anxiety might appear and further decrease the quality of life [36]. Particularly in mastocytosis, multiple mast cell degranulation symptoms and the fear of possible anaphylaxis probably contribute to this result. Nicoloro-SantaBarbara et al. [35] suggested that coping strategies work well in decreasing anxiety in patients with mast cell disorder. Little is known, however, about how anti-mediator therapy impacts anxiety.

The Quality of Life in Mastocytosis Scale (QLMS) has been validated with the Hospital Anxiety and Depression Scale, Cantril Ladder, STAI and Satisfaction with Life Scale by Spolak-Bobryk et al. [13] for use in patients with mast cell disorders. The questionnaire asks patients about their leisure time, protective behaviors, professional life and life limitations. The mean number 46.77 (with a range of 24–120) suggests a high quality of life; as in questionnaire scoring—the higher the score, the worse the outcome.

In our study, time of assessment was used as a covariate and did not impact the regression model [37]. The study was performed between January and July, and it is known that psychometric measures are influenced by seasonal changes. The authors mostly relate that connection to daylength, which has the most impact on mood and depression. The relationship to cognitive functions is uncertain as daylength influences particular domains differently. This phenomenon was also observed in a murine model showing variations in rats’ exploratory activity, and it appears to be connected to cholecystokinin and 5-HT2 receptors [38].

Although the exact mechanism of psychological disorders in mastocytosis remains unknown, some mast cell mediators still present an impact on nervous tissue. TNF increases the sensitivity of neurons, especially C fibers [39]. Tryptase may influence neurons through protease-activated receptors (PARs), particularly PAR2. This might lead to an increase in inflammation and hyperalgesia through the neurokinin 1 receptor [40].

All of the above suggest that psychological disfunctions play an important role in the course of mastocytosis’ clinical image. Thus, taking care of patients with mastocytosis proves to be multidisciplinary, involving dermatologists, hematologists, allergists and, in many cases, psychiatrists and gastroenterologists. This clearly shows how multifunctional mast cells in human body are. Future research might consider psychometric analysis in mast cell disorder evaluation and also look for different markers of cognitive impairment and depression in mastocytosis. Focusing on the Th1 profile released by mast cells might be another route.

## 4. Materials and Methods

### 4.1. Patient Recruitment and Clinical Evaluation

Patients were recruited randomly in the Polish Centre of Excellence in Mastocytosis in Gdansk between January 2023 and July 2023. Exclusion criteria were any history of cognitive disorder, aggressive mastocytosis, mast cell leukemia, lack of consent and age below 18. Questionnaire evaluation was completed in 115 subjects with MMSE. Furthermore, the study followed a screening paradigm for depression in mastocytosis that employed HDRS (performed by a certified rater) as the tool for patient mood assessment [19]. These questionnaires were followed by the Pruritus Visual Analog Scale (VAS), Quality of Life in Mastocytosis, State Trait Anxiety Inventory (STAI), serum tryptase and information on mast cell activation symptoms scored 0–3: flushing, diarrhea, cramping, stomach ache and headache. In 80 patients, serum was collected for cytokine analysis.

Archival bone marrow biopsy results were also collected with the assessment of: (1) KIT D816V mutation; (2) the presence of >15 mast cells per group in histopathology; (3) the expression of CD2, CD25 and CD30 on mast cells; and (4) the presence of >25% abnormal, spindle-shaped mast cells in bone marrow. Archival vein blood tests were also included: (1) complete blood count; (2) LDH; (3) fibrinogen; and (4) calcium.

The sample size was estimated at 80 based on a previous study by Spolak-Bobryk et al. using Altman’s Normogram with an alpha level of 0.05, a power of 0.8 and a standardized difference 0.6 on MMSE [13,41].

### 4.2. Sample Collection and Preparation

In order to obtain serum samples, venous blood was collected into a clot activator-containing tubes and allowed to clot for 30 min, followed by centrifugation for 15 min at 1000× *g*. Aliquoted serum samples were stored at −80 °C for further analyses. In all subjects that consented, blood was collected and after checking for stability all 80 samples were analyzed.

### 4.3. Determination of Cytokine Serum Levels

IL-4 serum levels were determined using the Human IL-4 ELISA kit (Antibodies.com, Cambridge, UK; cat. A78319) according to manufacturer’s instructions. The minimum detectable level of IL-4 was established as 18.75 pg/mL, with a 31.25–2000 pg/mL range. The intra-assay precision was estimated as CV < 8% and the inter-assay precision was estimated as CV < 10%. IL-31 serum levels were determined using the Human IL-31 ELISA kit (Antibodies.com, Cambridge, UK; cat. A76798) according to the manufacturer’s instructions. The minimum detectable level of the cytokine was established as 4.688 pg/mL. The range of the assay was established as 7.813–500 pg/mL. The intra-assay precision was estimated as CV < 8% and the inter-assay precision was estimated as CV < 10%. The serum levels of TSLP were determined using the Human TSLP ELISA kit (Antibodies.com, Cambridge, UK; cat. A78932) according to the manufacturer’s instructions. The minimum detectable level of the protein was established by the manufacturer as 18.75 pg/mL with a 31.25–2000 pg/mL range. The intra-assay precision was estimated as CV < 8% and the inter-assay precision was estimated as CV < 10%.

### 4.4. Statistical Analysis

Statistical analysis was performed with STATA 18 (StataCorp LLC, College Station, TX, USA). For comparison with continuous variables (MMSE, HDRS, STAI, Serum Tryptase, Pruritus VAS and cytokine concentration), simple linear regression was used. The results were adjusted for sex and age.

## 5. Conclusions

In mastocytosis, 26% of patients present cognitive disfunctions and 7.8% score below 24 points. The results of psychometric measures do not corelate significantly with IL-4, IL-31 and TSLP serum protein concentrations. However, this effect might be present when analyzing cytokine concentration on a tissue level or its RNA. Cognitive impairment evaluation should be considered in patients with mastocytosis in clinical and scientific practice. Future studies might consider evaluating other mast cell mediators in the context of psychometric evaluation; for example, the Th1 cytokine set.

## Figures and Tables

**Table 1:** Baseline characteristics of psychological evaluation of studied patient group. QLMS currently has no validated thresholds.

Evaluation	Mean (Points)	Standard Deviation	Number of Patients with Abnormal Result	Number of Patients with Normal Result
MMSE	27.9	2.62	28 (24%)	87 (76%)
HDRS	9.98	7.08	68 (59%)	47 (41%)
STAI-S	40.75	11.15	73 (63%)	42 (37%)
STAI-T	43.56	10.52	75 (65%)	40 (35%)
QLMS	46.77	21.47	–	–

**Table 2:** Results of linear regression between IL-4, IL-31, TSLP serum concentrations and study endpoints followed by tryptase serum concentration. MMSE—Mini-Mental State Examination; STAI-S—State Trait Anxiety Inventory for State; STAI-T—State Trait Anxiety Inventory for Trait; HDRS—17-item Hamilton Depression Rating Scale; QLMS—Quality of Life in Mastocytosis. All questionnaire results are one unit of change.

	Coefficient (95% Confidence Interval)			
Tested Cytokine	IL-4	IL-31	TSLP	Serum Tryptase
MMSE	−0.001 (−0.003–0.001)	−0.001 (−0.003–0.001)	0.001 (−0.001–0.003)	−0.006 (−0.016–0.005)
STAI-S	0.001 (−0.007–0.01)	−0.009 (−0.025–0.006)	0.003 (−0.004–0.013)	0.0319 (−0.013–0.076)
STAI-T	0.003 (−0.005–0.012)	−0.008 (−0.023–0.006)	0.002 (−0.007–0.01)	0.018 (−0.026–0.063)
HDRS	0.002 (−0.004–0.008)	−0.002 (−0.012–0.009)	−0.002 (−0.007–0.004)	0.001 (−0.029–0.031)
QLMS	−0.002 (−0.019–0.014)	−0.006 (−0.035–0.022)	0.011 (−0.005–0.028)	0.001 (−0.081–0.086)
Pruritus VAS	0.005 (−0.018–0.029)	−0.019 (−0.06–0.021)	0.016 (−0.007–0.0389)	0.007 (−0.006–0.021)
Serum Tryptase	−0.005 (−0.046–0.0349)	−0.001 (−0.07–0.068)	−0.005 (−0.045–0.034)	-
Mean Concentration in All Patients	412.9 pg/mL (STD dev 718.4)	204.9 pg/m (STD dev 415.7)	294.5 pg/mL (STD dev 675.3)	48 ng/mL (STD dev 47.5)

## Data Availability

Data are available on request.
